# Supplementary material for: Homeostatic Regulation of the Duox-ROS Defense System: Revelations Based on the Diversity of Gut Bacteria in Silkworms (Bombyx mori)
Source: Int J Mol Sci. 2023 Aug 12;24(16):12731. doi: 10.3390/ijms241612731 (PMC10454487; doi:10.3390/ijms241612731)
Supplement: Supplementary file 1 [file ijms-24-12731-s001.zip › Supplementary material S1.pdf]

Homeostatic regulation of the Duox-ROS defense system: rev-elations based on the diversity of gut bacteria in the silkworm (*Bombyx mori*)

Supplementary material

Table S1 Sequences of sequencing and qRT-PCR primers.

Gene	Forward primer (5'- 3')	Reverse primer (5'- 3')
27F-1429R	AGAGTTTGATCCTGGCTCAG	GGTTACCTTACGACTT
338F-806R	ACTCCTRCGGGAGGCAGCAG	GGACTACCVGGGTATCTAAT
RP49	CAGGCGGTTCAAGGGTCAATAC	TGCTGGGCTCTTTCCACGA
BmDuoX	CTCTACTCTGTTCCGATATACGA	CACCATTGTCTCTTCCTCTCA
BmArrestin	TTCGGTTCGGTTAGCAATCA	TGTTCTCGCCGTGGTAGT
BmMesh	GTATTCATTGCGTCAGGAGTG	TGGCTTCGTTGTAAGGTGTAA

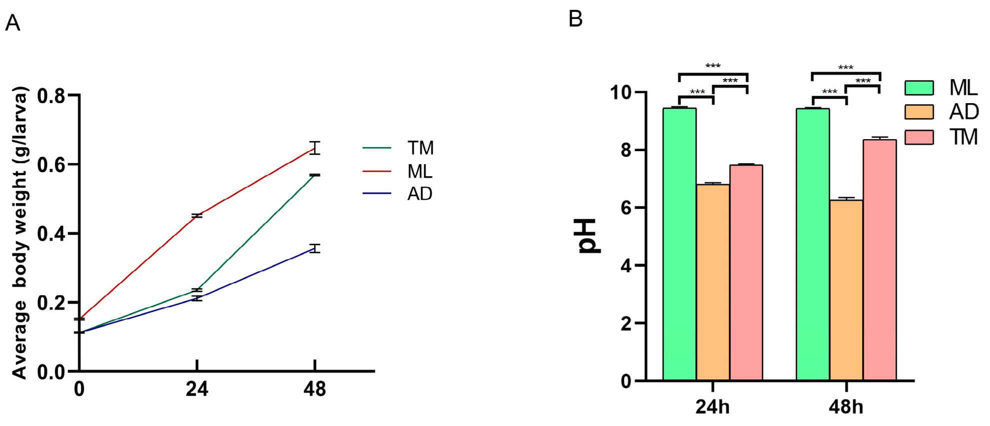

Supplementary Figure S1. Development of silkworm under different treatments. (A) Changes in body weight of silkworm during 0-48 h. (B) Changes in the intestinal pH of the silkworm during 0-48h. $p < 0.05$ (*), $p < 0.01$ (**), $p < 0.001$ (***), NS, Not Significant by Student's t-test.

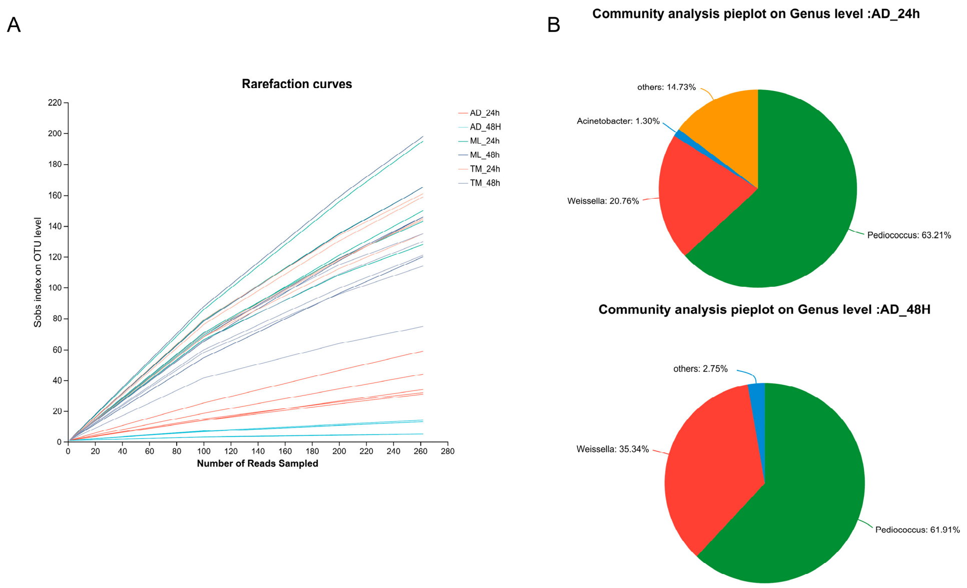

Supplementary Figure S2. Dilution curves of intestinal microbiota and genus level composition of colonies in the AD group of the silkworm. (A) Rarefaction curves of the samples. (B) Distribution of the genus level of the AD group of bacteria.

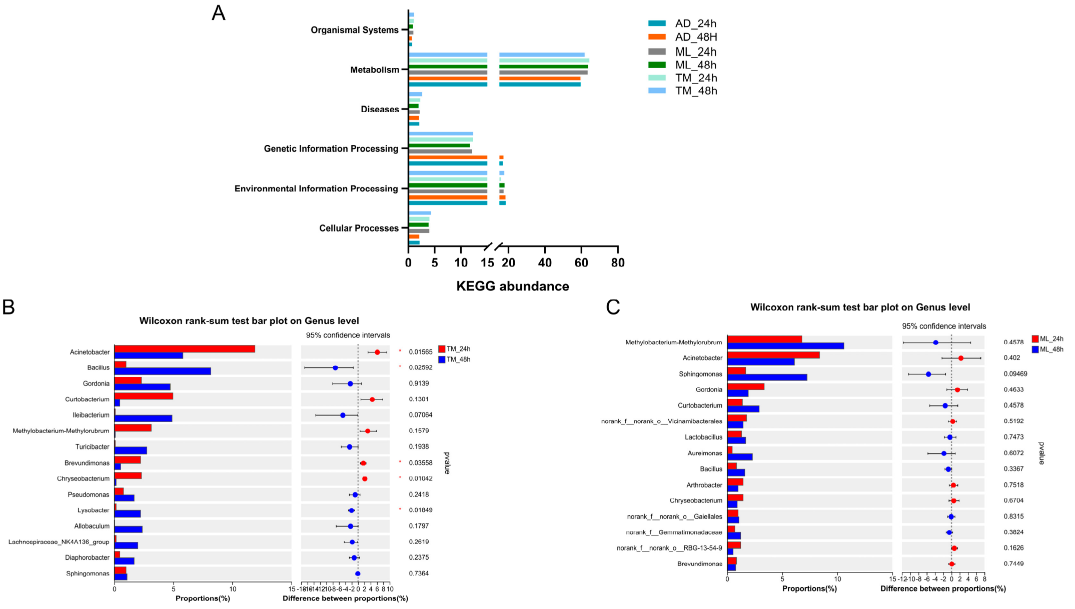

Supplementary Figure S3. Comparison of KEGG function prediction and differences within the gut microbiome. (A) KEGG function prediction. (B) Comparison of the differences between 24h and 48h intestinal microbiota in TM group. (C) Comparison of the differences between 24h and 48h microbiota in ML group. $p < 0.05$ (*), $p < 0.01$ (**), $p < 0.001$ (***), Student's t-test.
